# The Impact of Endometriosis on Embryo Quality in *in-vitro* Fertilization/Intracytoplasmic Sperm Injection: A Systematic Review and Meta-Analysis

**DOI:** 10.3389/fmed.2021.669342

**Published:** 2021-06-02

**Authors:** Houjin Dongye, Xiaofeng Ji, Xiaopei Ma, Jialun Song, Lei Yan

**Affiliations:** ^1^Center for Reproductive Medicine, Cheeloo College of Medicine, Shandong University, Jinan, China; ^2^Key Laboratory of Reproductive Endocrinology of Ministry of Education, Shandong University, Jinan, China; ^3^Shandong Key Laboratory of Reproductive Medicine, Jinan, China; ^4^Shandong Provincial Clinical Research Center for Reproductive Health, Jinan, China; ^5^National Research Center for Assisted Reproductive Technology and Reproductive Genetics, Shandong University, Jinan, China; ^6^Department of Gynecology, The Eighth People's Hospital of Xinjiang Uygur Autonomous Region, Urumqi, China

**Keywords:** endometriosis, embryo quality, morphological evaluation, IVF, ICSI

## Abstract

**Background:** The association between endometriosis and embryological outcomes remains uncertain. The meta-analysis aimed to evaluate the impact of endometriosis on embryo quality.

**Methods:** A systematic review and meta-analysis was conducted to investigate the association between the endometriosis and embryo quality. Searches were performed on the three electronic databases: PubMed, EMBASE, and Web of Science. The detailed characteristics and data of the included studies were extracted. The risk ratio with 95% confidence intervals were calculated using the random and fixed effects model. The main outcome measures were high-quality embryo rate, cleavage rate, and embryo formation rate.

**Results:** A total of 22 studies included were analyzed. Compared with the control group, women with endometriosis had a similar high-quality embryo rate (RR = 1.00; 95% CI, 0.94–1.06), a comparable cleavage rate (RR = 1.00; 95% CI, 0.97–1.02), and a similar embryo formation rate (RR = 1.10; 95% CI, 0.97–1.24). In women with stage III-IV endometriosis, there was no statistically significantly difference in high-quality embryo rate (RR = 1.02; 95% CI, 0.94–1.10), cleavage rate (RR = 1.00; 95% CI, 0.98–1.02), and embryo formation rate (RR = 1.05; 95% CI, 0.97–1.14), compared with those without endometriosis. For women with unilateral endometrioma, pooling of results from the affected ovaries did not show a statistically significantly difference in high-quality embryo rate (RR = 0.99; 95% CI, 0.60–1.63) in comparison to the normal contralateral ovaries.

**Conclusions:** Our results seem to indicate that endometriosis does not compromise embryo quality from the perspective of morphology.

## Introduction

Endometriosis, wherein endometrial tissue including glands and stroma is present outside the uterine cavity, affects ~10% of women in reproductive age and 40% of women with infertility (1, 2). Studies have shown that endometriosis has adverse effect on fertility in reproductive women. The corresponding mechanisms mainly include reduction of functional ovarian tissue resulted from endometriomas or surgery, inflammatory changes in peritoneal fluid, reduction in endometrial receptivity and alteration in the number and quality of oocyte or embryo. However, the explicit causes are still poorly understood (3). Assisted reproductive technology (ART) is an effective approach for endometriosis-associated infertility (4).

Many research papers pertaining to the consequence of endometriosis on the outcomes of ART have been published; nevertheless, these results remain still controversial (5–7). More specifically, it also seemed disputed in terms of the association between endometriosis and embryological outcomes (8–10). This respect is essential considering that efforts are made to select high-quality embryos for transfer in embryological laboratories, especially, elective single-embryo transfer (eSET) has been increasingly advocated to reduce the risk of multiple gestations and improve pregnancy outcomes (11). It is of significance to further elucidate this aspect, as so far with conflicting, and to the best of our knowledge, no meta-analysis specifically focusing on the association between endometriosis and embryological outcomes is present. The aims of our systematic review and meta-analysis are to investigate the association between endometriosis and embryological outcomes from the morphological perspective and further review whether the severity of endometriosis or unilateral endometrioma has a negative effect on embryo formation and development.

## Methods

### Search Strategy and Selection Criteria

PubMed, EMBASE, and Web of Science were searched by two independent reviewers using the keywords and/or medical subject heading (MeSH) terminology: endometriosis, endometrioma, ART, *in-vitro* fertilization, and intracytoplasmic sperm injection, embryo. The final search was performed in August 2020.

The inclusion criteria were as follows: ① cohort studies (retrospective or prospective); ② women underwent *in-vitro* fertilization/intracytoplasmic sperm injection (IVF/ICSI); ③ study group consisted of women with endometriosis diagnosed by laparoscopy, histology, ultrasound, or magnetic resonance imaging (MRI); ④ women with or without prior treatment (surgery or medicine) for endometriosis; ⑤ control study were women without endometriosis including those with tubal infertility, male factor infertility, unexplained infertility or mixed etiology infertility; ⑥ the embryo at cleavage stage were assessed morphologically.

The exclusion criteria included: ① non-English papers; ② studies without a control group; ③ literatures such as conference abstracts or other personal communication; ④ women with diseases such as polycystic ovary syndrome (PCOS) and premature ovarian failure, which may cause damage to embryo; ⑤ women involved with donor or recipient oocytes treatment.

### Data Extraction and Quality Assessment

The primary outcome was a high-quality embryo rate. The secondary outcomes were cleavage rate and embryo formation rate. After an initial screen of all titles and abstracts retrieved from the electronic searches, the full texts of all potentially eligible studies were obtained. Two reviewers respectively scrutinized these articles to select the papers qualified for aforementioned inclusion criteria. Disagreements were resolved through discussions with a third reviewer. Two reviewers independently extracted the outcome data and study characteristics using a specifically designed form. These data were examined repeatedly by both investigators. Discrepancies were resolved by discussion with consensus.

The assessment of study quality was implemented by two reviewers based on the Newcastle-Ottawa Quality Assessment Scale for observational studies. The scale involves eight items categorized in three domains: selection, comparability, and outcome, with each item can be awarded a maximum of one star, except comparability, which can be given up to two stars. Eventually, results presented as the number of stars ranging from one to nine. We performed analyses in studies where embryological outcomes in women with endometriosis or stage III-IV endometriosis, which were classified according to the rAFS/ASRM (revised classification of the American Fertility Society or Revised American Society for Reproductive Medicine classification of endometriosis), were compared with those without endometriosis. Additionally, we compared embryological outcomes between affected ovary and intact ovary in women with unilateral endometrioma. The systematic review and meta-analysis were reported in accordance with the Meta-analysis of Observational Studies in Epidemiology (MOOSE) statement.

### Statistical Analysis

The statistical analysis was performed using Review Manager version 5.4. Relevant data was abstracted from original papers, if not presented, then calculated by using matching raw data provided. For dichotomous variables, results for each indicator were expressed as risk ratio (RR) with 95% confidence intervals (CIs). Funnel plots were constructed to assess the publication bias. Sensitivity analyses were completed in the means of removing one of included studies at a time from the meta-analysis and recalculating the combined effect size to evaluate the effect of every study on the pooled effect size.

The *I*^2^ were measured to quantify statistical heterogeneity. A fixed effects model was used when *I*^2^ < 50%, while a random effects model of DerSimonian and Laird was applied if *I*^2^ ≥ 50%. A random effects model implied the effects analyzed among the included studies were not identical but followed similar distributions.

## Results

### Study Selection and Characteristics

**Figure 1:**
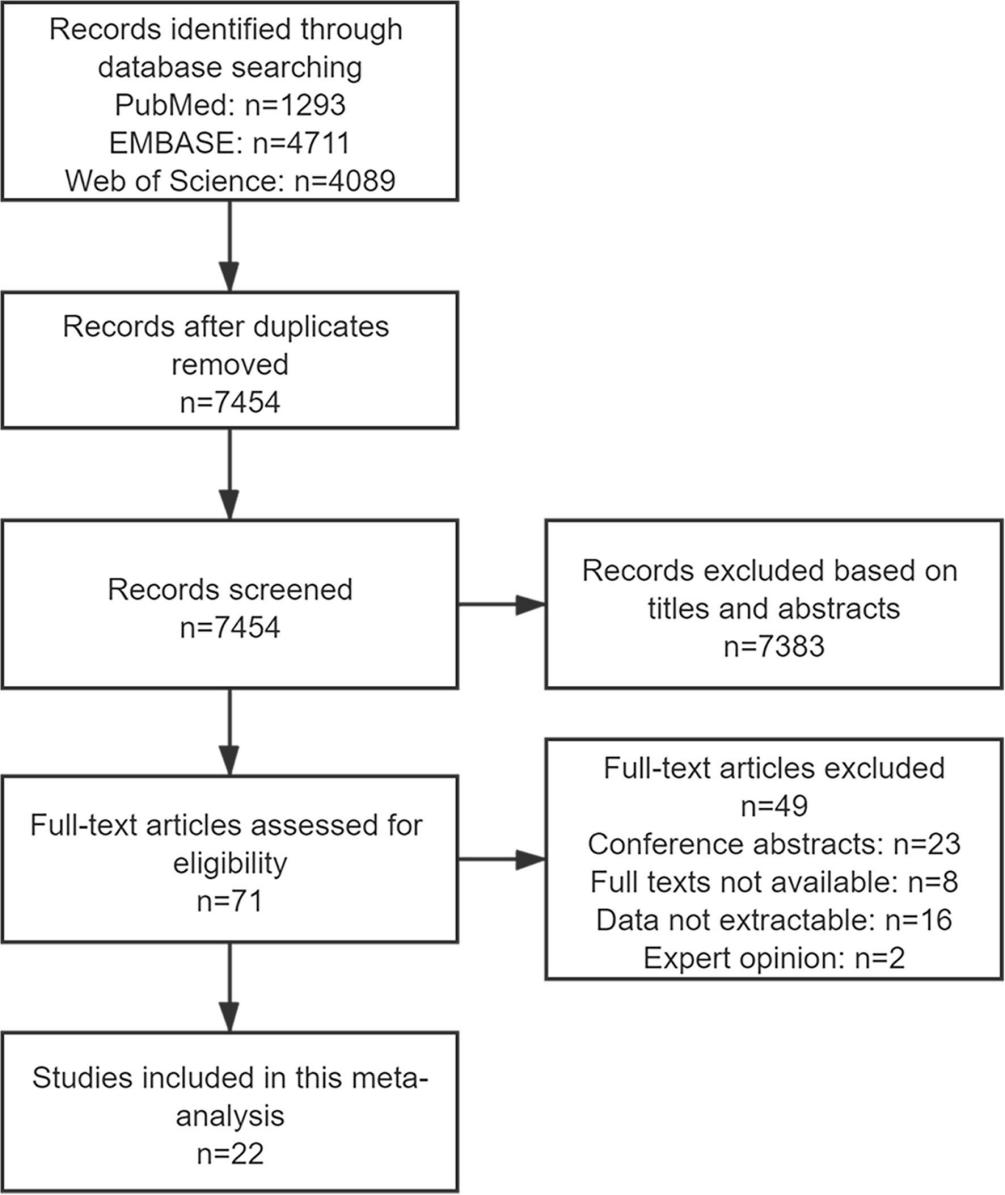
Flow diagram for study selection process.

The search strategy retrieved 1,293 citations from PubMed, 4,711 from EMBASE, and 4,089 from Web of Science. The duplicate studies were removed, after importing all results into EndNote, leaving 7,454 articles. With regard to duplicate publication, only the most recent and complete versions were chosen. A total of 7,383 records were excluded following an initial screening for the titles and abstracts. After reviewing full texts of the remaining 71 studies, 49 articles were excluded for conference abstracts (*n* = 23), full texts not available (*n* = 8), data not extractable (*n* = 16), and expert opinion (*n* = 2). Therefore, a number of 22 publications were eligible for selection criteria and included in the review (Figure 1).

With regard to the study design, all the included studies were observational studies, and 22 were cohort studies (six prospective, 14 retrospective). Of these included studies, the study groups contained endometriosis (*n* = 17), stage III-IV endometriosis (*n* = 12), and unilateral endometrioma (*n* = 3). The diagnosis of endometriosis was based on laparoscopy (*n* = 13), histology (*n* = 2), and ultrasound (*n* = 8). In 22 of the included studies, the stage of endometriosis was performed on the basis of the rAFS/ASRM. Studies evaluated the embryo quality in women with endometriosis or stage III-IV endometriosis and included various control groups: tubal infertility (*n* = 9), male factor infertility (*n* = 6), unexplained infertility (*n* = 1), and mixed etiology infertility (*n* = 5). The control groups were drawn from the same community or hospital as the study groups. The detailed characteristics of the included studies are presented in Supplementary Table 1.

### Quality Assessment of Included Studies

The majority of included studies (*n* = 12) were awarded eight stars, two studies were awarded nine stars, six studies were awarded seven stars, and only two studies scored six stars. The Newcastle-Ottawa Quality Assessment Scale is shown in Supplementary Table 2.

### Synthesis of Results

**Figure 2:**
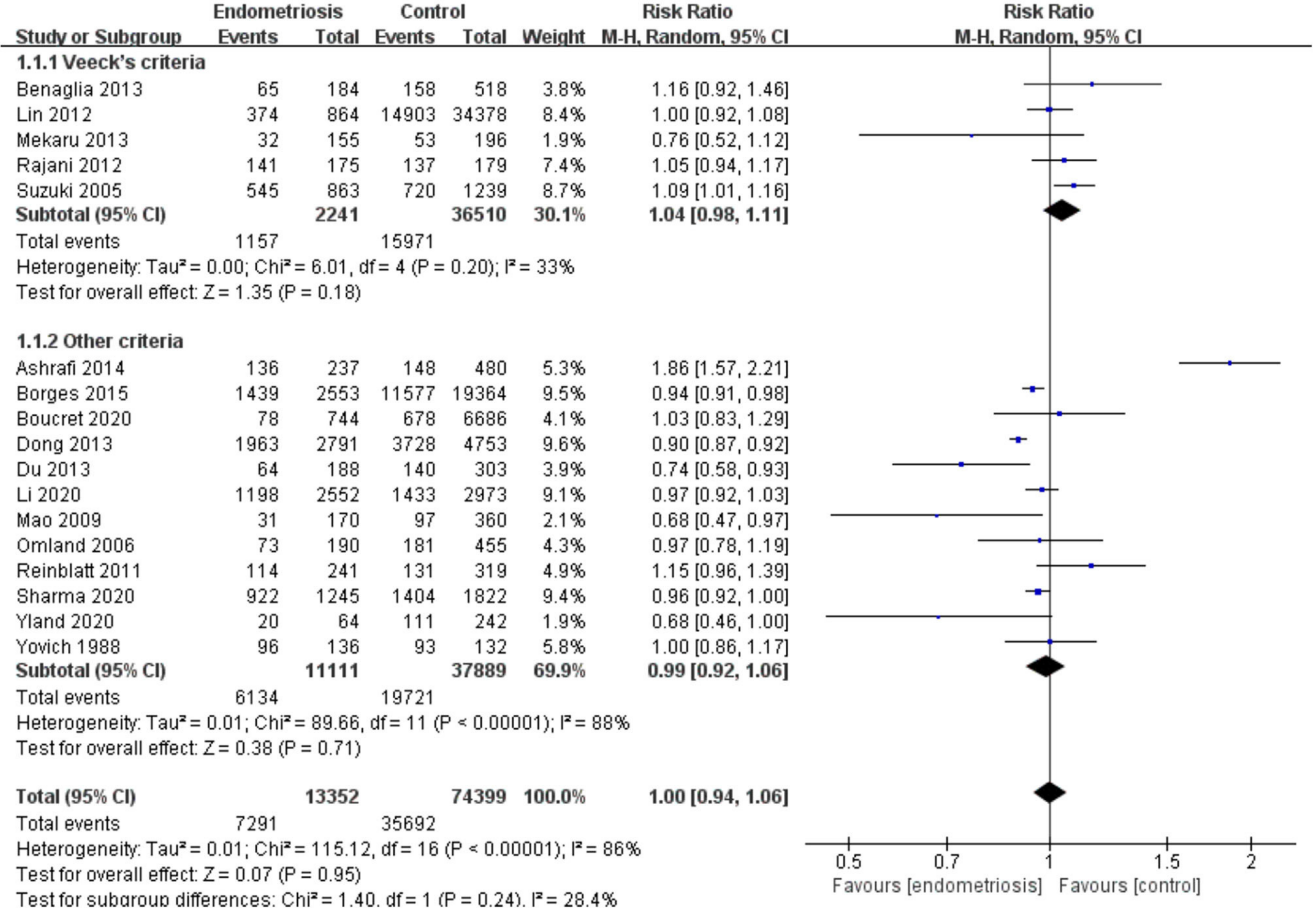
Forest plot of high-quality embryo rate for endometriosis vs. control.

**Figure 3:**
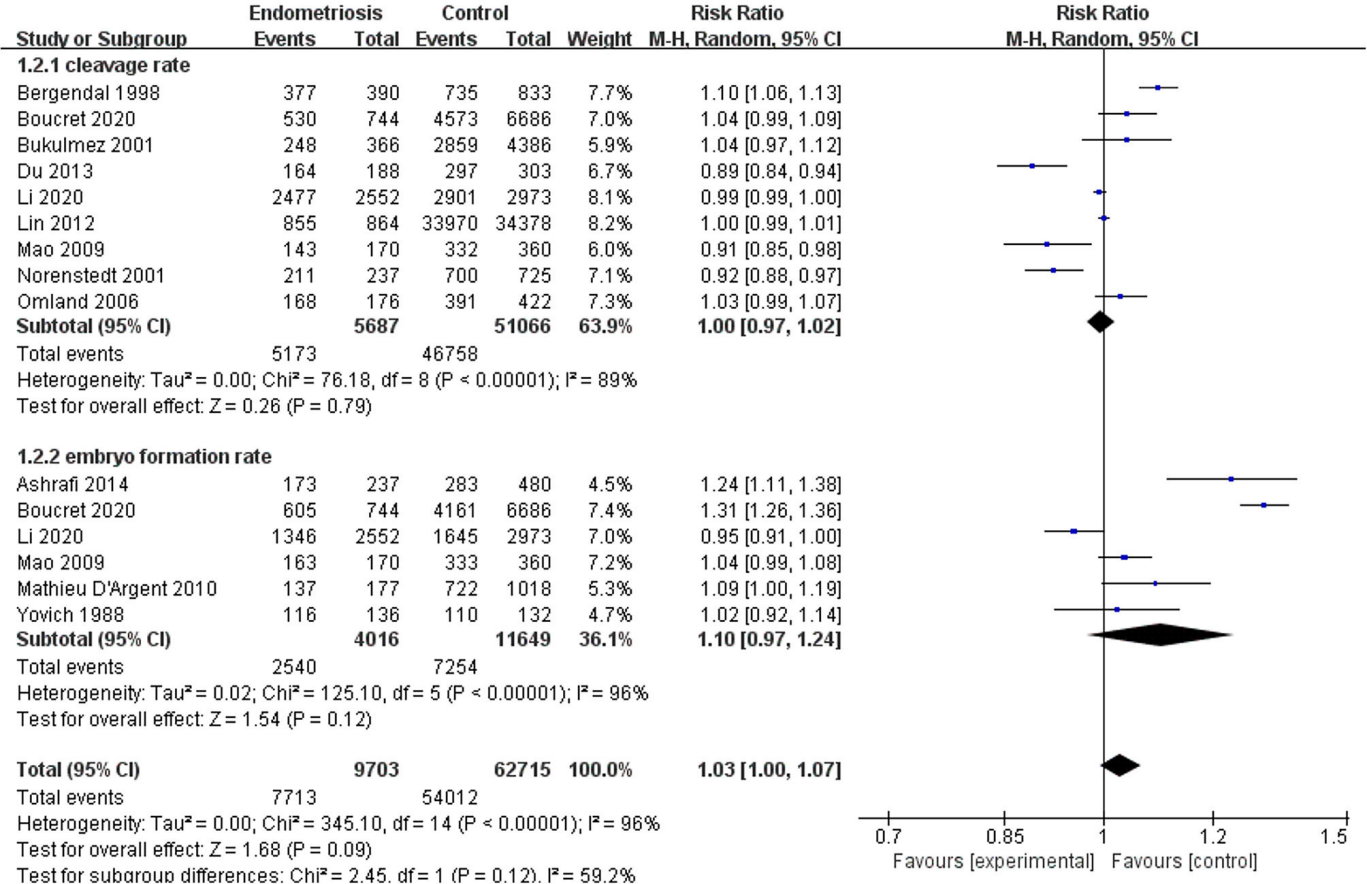
Forest plot of cleavage rate and embryo formation rate for endometriosis vs. control.

**Figure 4:**
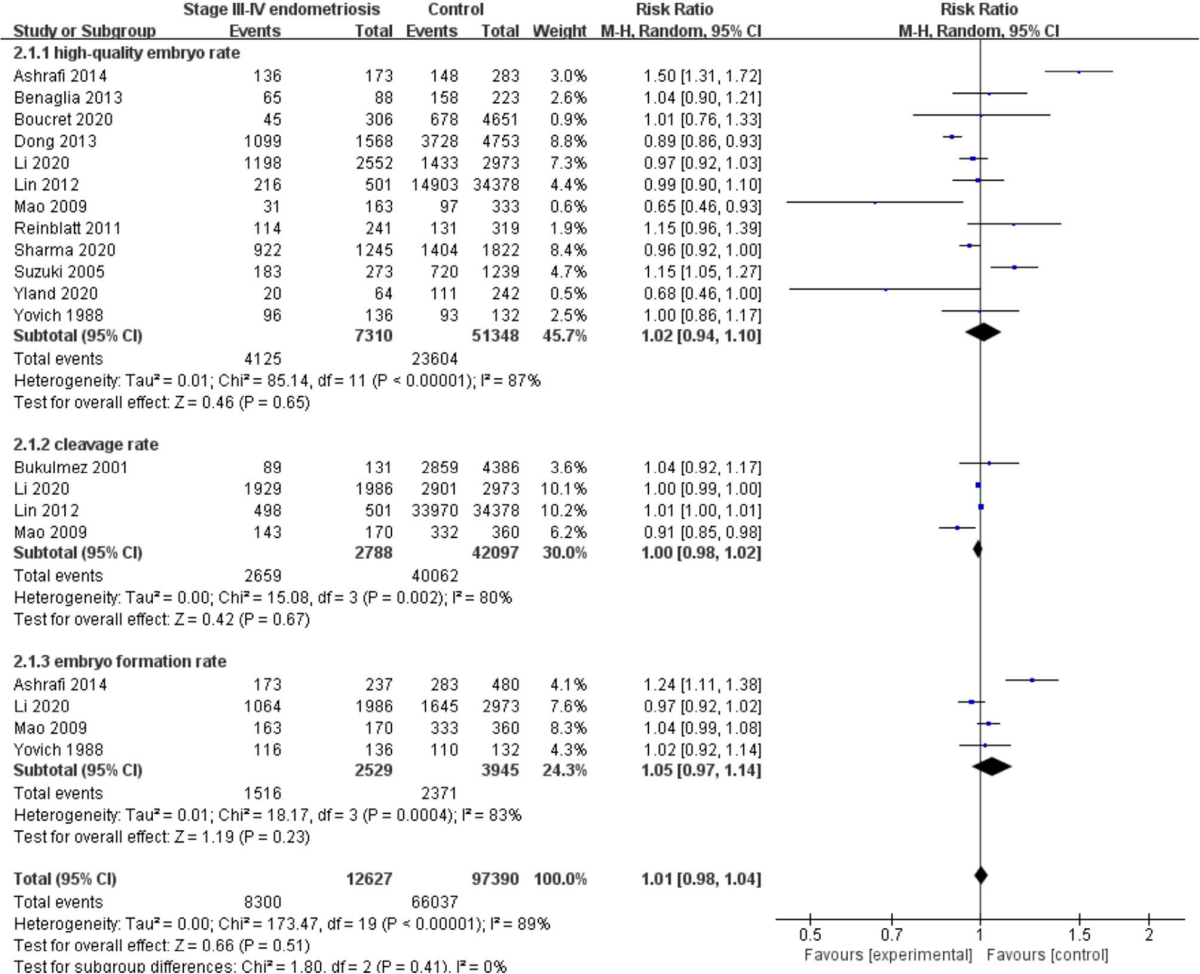
Forest plot of high-quality embryo rate, cleavage rate, and embryo formation rate for stage III-IV endometriosis vs. control.

**Figure 5:**
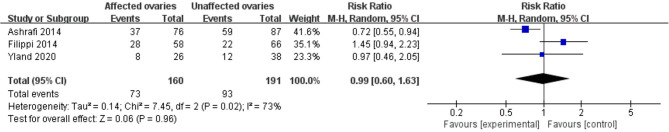
Forest plot of high-quality embryo rate for affected ovaries vs. unaffected ovaries.

Compared with the control group, women with endometriosis had a similar high-quality embryo rate (RR = 1.00; 95% CI, 0.94–1.06) (Figure 2). No differences were found in cleavage rate (RR = 1.00; 95% CI, 0.97–1.02) and embryo formation rate (RR = 1.10; 95% CI, 0.97–1.24) (Figure 3). In women with stage III-IV endometriosis, there was no statistically significantly difference in high-quality embryo rate (RR = 1.02; 95% CI, 0.94–1.10), compared with those without endometriosis. Other indicators such as cleavage rate (RR = 1.00; 95% CI, 0.98–1.02) and embryo formation rate (RR = 1.05; 95% CI, 0.97–1.14) were also comparable between both groups (Figure 4). For women with unilateral endometrioma, pooling of results from the affected ovaries did not show a statistically significantly difference in high-quality embryo rate (RR = 0.99; 95% CI, 0.60–1.63) in comparison to the normal contralateral ovaries (Figure 5).

## Discussion

### Main Findings

We found no statistically significantly differences in high-quality embryo rate, cleavage rate, and embryo formation rate in women with endometriosis compared with those without endometriosis. Moreover, the aforementioned indicators were comparable between women with severe endometriosis (stage III-IV) and those without endometriosis. In addition, results from both affected ovaries and intact ovaries were similar.

### Strengths and Limitations

To our knowledge, no previous systematic review and meta-analysis concerning the association between endometriosis and embryo quality is as large scale, up to date, and comprehensive. A prior meta-analysis by Yang et al. have reported a lower number of total embryos formed and a similar number of good-quality embryos between women with endometrioma and control group. They also have made comparisons between ovaries affected and normal contralateral ovaries; no difference was shown in the number of total embryos formed (12). However, the results need to be interpreted with caution due to only two included studies and the two observed indicators, which were not recommended by the Vienna consensus (13). There are also some limitations to be noted in this review. First, we only included published English studies, thus non-English studies as well as conference abstracts were excluded, which may result in selection bias. Second, some issues remain in both comparison groups. The concordance among control groups is not satisfactory because the causes of infertility in control group vary between tubal and male factor, even several studies just including non-endometriosis women not limiting to specific etiologies. It is also worth noting that these etiologies may have an adverse influence on embryo quality respectively or collectively (14). The same is true in endometriosis groups, in which whether to receive treatment or not and which therapeutic modality was performed are not well-controlled. Suzuki et al. reported that their data indicated the negative effect of endometriosis could be compromised by laparoscopic treatment (15). Finally, so far, no consensus on embryo morphological assessment has been applied worldwide, albeit embryologists are dedicated to selecting embryos for transfer based on morphological features. This is a major disadvantage because the differences in the criteria for evaluating embryo quality may compromise the homogeneity between included studies. Nevertheless, the majority of embryo grading systems existing mainly take into consideration the following indexes: the number and symmetry of blastomeres, the relative degrees of fragmentation, and the presence or absence of multinucleation (16), which, to some extent, could reduce the heterogeneity of interstudies.

### Interpretation and Implication

Currently, it is extremely difficult to draw a definite conclusion on the association between endometriosis and embryo quality, with results controversial. A number of studies have suggested that endometriosis has a detrimental impact on embryo quality (17–20), while some studies are unable to demonstrate the relationship between the two (9, 10, 15, 21). Lin et al. showed a lower high-quality embryo rate in an endometriosis group including 177 women compared with the control group comprising the remaining 4,267 women with any factors other than endometriosis through collecting information from the electronic records between January 2006 and December 2010 in their hospital (19). Further studies observed elevated levels of inflammatory cytokines in both follicular and peritoneal fluid, such as interleukin-6 (IL-6), IL-8, and tumor necrosis factors (22, 23). The alteration of the follicular and peritoneal microenvironment may not be conducive to the development and maturation of oocytes, and subsequently, may potentially affect embryo development (24).

Our results do not seem to support the adverse effect of endometriosis on embryo quality. These following reasons could account for our findings. A successful pregnancy requires not only high-quality embryos but also a receptive endometrium. Emerging evidences indicate that inflammation plays a vital role in the pathogenic mechanisms of endometriosis (25). Endometriosis induces a series of local and systemic inflammatory responses, and these disordered inflammatory cytokines subsequently interfere with normal endometrial function through complex signal pathways, eventually, leading to less-receptive endometrium for embryo implantation (26). Another reason, namely, the limitations of conventional embryos morphological assessment, is also of great importance. The common indicators of embryo morphological evaluation may not reflect the intrinsic changes of embryos retrieved from women with endometriosis well; that is, the effect of endometriosis on embryos may not be presented as the altered morphology (27). Consequently, it is possible that grading embryos in light of morphological features is imprecise (28). Remarkably, in this review, a higher yet not statistically significant high-quality embryo rate was observed in the endometriosis group than the control group according to Veeck's criteria. An embryo scoring scheme, which considers various factors not limiting to morphological features, is required.

## Conclusions

Our results indicate that endometriosis does not compromise embryo quality from the perspective of morphology. The universal criteria and terminology for grading embryos are required to reduce the heterogeneity between studies, thus making comparisons of clinical data in this field more statistically powerful. More high-quality, well-designed research with a large sample size as well as population strictly selected need to be undertaken to elucidate the association between endometriosis (especially its subtypes and stages) and embryo quality.

## Data Availability Statement

All datasets generated for this study are included in the article/Supplementary Material, further inquiries can be directed to the corresponding author/s.

## Author Contributions

HD, JS, and LY conceived and designed the study. XJ performed the literature search. XM extracted the data. HD analyzed the data and wrote the manuscript. LY and JS revised the manuscript and supervised the study. All authors read and approved the final manuscript.

## Conflict of Interest

The authors declare that the research was conducted in the absence of any commercial or financial relationships that could be construed as a potential conflict of interest.
