# Supplementary material for: The Impact of Endometriosis on Embryo Quality in in-vitro Fertilization/Intracytoplasmic Sperm Injection: A Systematic Review and Meta-Analysis
Source: Front Med (Lausanne). 2021 Jun 2;8:669342. doi: 10.3389/fmed.2021.669342 (PMC8206501; doi:10.3389/fmed.2021.669342)
Supplement: Supplementary file 1 [file Table_1.DOCX]

**Supplementary Table 1**. Characteristics of the included studies

| **Author** | **Year** | **Location** | **Duration** | **Design** | **Diagnosis of endometriosis** |
| --- | --- | --- | --- | --- | --- |
| **Ashrafi et al.** | 2014 | Iran | 2005-2007 | Prospective cohort study | Ultrasound |
| **Benaglia et al.** | 2013 | Italy | 2006-2010 | Retrospective cohort study | Ultrasound |
| **Bergendal et al.** | 1998 | Sweden | 1994-1997 | Retrospective cohort study | Laparoscopy, biopsy |
| **Borges et al.** | 2015 | Brazil | 2005-2014 | Single central cohort study | Laparoscopy |
| **Boucret et al.** | 2020 | France | 2014-2018 | Retrospective cohort study | Ultrasound, MRI |
| **Bukulmez et al.** | 2001 | Turkey | 1996-1999 | Retrospective cohort study | Laparoscopy |
| **Dong et al.** | 2013 | China | 2011-2012 | Retrospective cohort study | Laparoscopy |
| **Du et al.** | 2013 | China | 2010 | Prospective cohort study | Laparoscopy |
| **Filippi et al.** | 2014 | Italy | 2012-2013 | Prospective cohort study | Ultrasound |
| **Li et al.** | 2020 | China | 2016-2017 | Retrospective cohort study | Laparoscopy, ultrasound |
| **Lin et al.** | 2012 | China | 2006-2010 | Retrospective cohort study | Laparoscopy, laparotomy |
| **Mao et al.** | 2009 | China | 2003-2006 | Retrospective cohort study | Laparoscopy |
| **Mathieu et al.** | 2010 | France | 2007-2008 | Retrospective cohort study | Ultrasound, MRI |
| **Mekaru et al.** | 2013 | Japan | 2004-2008 | Retrospective cohort study | Laparoscopy |
| **Norenstedt et al.** | 2001 | Sweden | 1995-1999 | Retrospective cohort study | Laparoscopy, biopsy |
| **Omland et al.** | 2006 | Norway | 1997-2006 | Retrospective cohort study | Laparoscopy |
| **Rajani et al.** | 2012 | India | Not reported | Prospective cohort study | Not reported |
| **Reinblatt et al.** | 2011 | Canada | 2006-2010 | Retrospective cohort study | Ultrasound |
| **Sharma et al.** | 2020 | India | 2012-2018 | Prospective cohort study | Laparoscopy |
| **Suzuki et al.** | 2005 | Japan | 1996-2002 | Retrospective cohort study | Laparoscopy |
| **Yland et al.** | 2020 | USA | 2012-2013 | Prospective cohort study | Laparoscopy, ultrasound |
| **Yovich et al.** | 1988 | Australia | Not reported | Cohort study | Laparoscopy |

**Supplementary Table 1**. Continued

| **Author** | **Study group** | **N** | **Cycles** | **Control group** | **N** | **Cycles** | **Criteria for evaluating embryo quality** |
| --- | --- | --- | --- | --- | --- | --- | --- |
| **Ashrafi et al.** | Endometrioma | 47 | Not reported | Mild male factor | 57 | Not reported | Baczkowski T 2004 |
| **Benaglia et al.** | Bilateral endometriomas | 39 | Not reported | Male factor, tubal factor, anovulatory, unexplained | 78 | Not reported | Veeck’s criteria |
| **Bergendal et al.** | Endometriosis | 48 | 65 | Tubal factor | 98 | 98 | Self-defined |
| **Borges et al.** | Endometriosis | Not reported | 431 | Mixed etiology infertility | Not reported | 2510 | Self-defined |
| **Boucret et al.** | Endometriosis, stage I-II, stage III-IV | 84 | 155 | Male factor, prior fertilization failure | 590 | 969 | Istanbul consensus 2011 |
| **Bukulmez et al.** | Endometriosis, stage I-II, stage III-IV | 44 | 78 | Male factor | 588 | 895 | Self-defined |
| **Dong et al.** | Endometriosis, stage I-II, stage III-IV | Not reported | 431 | Tubal factor | Not reported | 596 | Self-defined |
| **Du et al.** | Stage II-III | 29 | Not reported | Male factor | 36 | Not reported | Self-defined |
| **Filippi et al.** | Unilateral endometrioma (affected ovary) | 29 | Not reported | Normal contralateral ovary | 29 | Not reported | Istanbul consensus 2011 |
| **Li et al.** | Stage III-IV | 459 | 442 | Tubal factor | 360 | 474 | Cummins JM 1986 |
| **Lin et al.** | Endometriosis | 177 | Not reported | Tubal factor, male factor, complex factors | 4267 | Not reported | Veeck’s criteria |
| **Mao et al.** | Endometrioma, stage IV | 32 | 71 | Tubal factor | 32 | 59 | WIH score system |
| **Mathieu et al.** | Colorectal endometriosis | 29 | Not reported | Tubal factor | 257 | Not reported | Scott L 2000 |
| **Mekaru et al.** | Stage I-II | 18 | 39 | Unexplained infertility | 17 | 41 | Veeck’s criteria |
| **Norenstedt et al.** | Endometriosis | 26 | 43 | Male factor | 125 | 125 | Fridstrom M 1995 |
| **Omland et al.** | Stage I | Not reported | 43 | Male factor | Not reported | 91 | Van Den Abbeel E 1988 |
| **Rajani et al.** | Endometriosis | 56 | Not reported | Tubal factor | 63 | Not reported | Veeck’s criteria |
| **Reinblatt et al.** | Bilateral endometriomas | 13 | Not reported | Male or tubal factor | 39 | Not reported | Self-defined |
| **Sharma et al.** | Stage III-IV | 294 | Not reported | Tubal factor | 358 | Not reported | Self-defined |
| **Suzuki et al.** | Endometriosis, endometrioma | Not reported | 328 | Tubal factor | Not reported | 283 | Veeck’s criteria |
| **Yland et al.** | Unilateral endometrioma | 10 | Not reported | Male factor | 24 | Not reported | Racowsky C 2003 |
| **Yovich et al.** | Stage IV pelvic endometriosis | 30 | Not reported | Tubal factor | 28 | Not reported | Yovich JL 1985 |
