# Supplementary material for: The Impact of Endometriosis on Embryo Quality in in-vitro Fertilization/Intracytoplasmic Sperm Injection: A Systematic Review and Meta-Analysis
Source: Front Med (Lausanne). 2021 Jun 2;8:669342. doi: 10.3389/fmed.2021.669342 (PMC8206501; doi:10.3389/fmed.2021.669342)
Supplement: Supplementary file 2 [file Table_2.DOCX]

**Supplementary Table 2**. The Newcastle-Ottawa Quality Assessment Scale

| **Author** | **Year** | **Representativeness of the exposed cohort** | **Selection of the non-exposed cohort** | **Ascertainment of exposure** | **Demonstration that outcome of interest was not present at start of study** | **Comparability of cohorts on the basis of the design or analysis** | **Assessment of outcome** | **Was follow-up long enough for outcomes to occur** | **Adequacy of follow up of cohorts** | **Score** |
| --- | --- | --- | --- | --- | --- | --- | --- | --- | --- | --- |
| **Ashrafi et al.** | 2014 | * | * | * | * | ** |  | * | * | 8 |
| **Benaglia et al.** | 2013 | * | * | * |  | ** | * | * | * | 8 |
| **Bergendal et al.** | 1998 | * | * | * |  | * | * | * | * | 7 |
| **Borges et al.** | 2015 | * | * |  |  | ** |  | * | * | 6 |
| **Boucret et al.** | 2020 | * | * | * |  | ** | * | * | * | 8 |
| **Bukulmez et al.** | 2001 | * | * | * |  | ** | * | * | * | 8 |
| **Dong et al.** | 2013 | * | * | * |  | ** | * | * | * | 8 |
| **Du et al.** | 2013 | * | * | * | * | * | * | * | * | 8 |
| **Filippi et al.** | 2014 | * | * | * | * | ** | * | * | * | 9 |
| **Li et al.** | 2020 | * | * | * |  | ** | * | * | * | 8 |
| **Lin et al.** | 2012 | * | * | * |  | ** | * | * | * | 8 |
| **Mao et al.** | 2009 | * | * | * |  | ** | * | * | * | 8 |
| **Mathieu et al.** | 2010 | * | * | * |  | * | * | * | * | 7 |
| **Mekaru et al.** | 2013 | * | * | * |  | * | * | * | * | 7 |
| **Norenstedt et al.** | 2001 | * | * | * |  | * | * | * | * | 7 |
| **Omland et al.** | 2006 | * | * | * |  | * | * | * | * | 7 |
| **Rajani et al.** | 2012 | * | * |  | * | * | * | * | * | 7 |
| **Reinblatt et al.** | 2011 | * | * | * |  | ** | * | * | * | 8 |
| **Sharma et al.** | 2020 | * | * | * | * | ** |  | * | * | 8 |
| **Suzuki et al.** | 2005 | * | * | * |  | ** | * | * | * | 8 |
| **Yland et al.** | 2020 | * | * | * | * | ** | * | * | * | 9 |
| **Yovich et al.** | 1988 | * | * |  |  | * | * | * | * | 6 |
